# Caesarean birth rates in public and privately funded hospitals: a cross-sectional study

**DOI:** 10.11606/S1518-8787.2017051007054

**Published:** 2017-11-13

**Authors:** Bruna Dias Alonso, Flora Maria Barbosa da Silva, Maria do Rosário Dias de Oliveira Latorre, Carmen Simone Grilo Diniz, Debra Bick

**Affiliations:** I Universidade de São Paulo. Faculdade de Saúde Pública. Programa de Pós-Graduação em Saúde Pública. São Paulo, SP, Brasil; II Universidade de São Paulo. Faculdade de Saúde Pública. Escola de Artes, Ciências e Humanidades. Departamento de Saúde Materno-Infantil. Graduação em Obstetrícia. São Paulo, SP, Brasil; III Universidade de São Paulo. Faculdade de Saúde Pública. Departamento de Epidemiologia. São Paulo, SP, Brasil; IV Universidade de São Paulo. Faculdade de Saúde Pública. Departamento de Saúde Materno-Infantil. São Paulo, SP, Brasil; V King’s College London. Florence Nightingale Faculty of Nursing and Midwifery. London, United Kingdom

**Keywords:** Cesarean Section, statistics & numerical data, Health care Financing, Maternal-Child Health Services, Socioeconomic Factors, Cross-Sectional Studies

## Abstract

**OBJECTIVE:**

To examine maternal and obstetric factors influencing births by cesarean section according to health care funding.

**METHODS:**

A cross-sectional study with data from Southeastern Brazil. Caesarean section births from February 2011 to July 2012 were included. Data were obtained from interviews with women whose care was publicly or privately funded, and from their obstetric and neonatal records. Univariate and multivariate analyses were conducted to generate crude and adjusted odds ratios (OR) with 95% confidence intervals (95%CI) for caesarean section births.

**RESULTS:**

The overall caesarean section rate was 53% among 9,828 women for whom data were available, with the highest rates among women whose maternity care was privately funded. Reasons for performing a c-section were infrequently documented in women’s maternity records. The variables that increased the likelihood of c-section regardless of health care funding were the following: paid employment, previous c-section, primiparity, antenatal and labor complications. Older maternal age, university education, and higher socioeconomic status were only associated with c-section in the public system.

**CONCLUSIONS:**

Higher maternal socioeconomic status was associated with greater likelihood of a caesarean section birth in publicly funded settings, but not in the private sector, where funding source alone determined the mode of birth rather than maternal or obstetric characteristics. Maternal socioeconomic status and private healthcare funding continue to drive high rates of caesarean section births in Brazil, with women who have a higher socioeconomic status more likely to have a caesarean section birth in all birth settings.

## INTRODUCTION

High rates of caesarean section (CS) are a public health issue. Overall CS rates higher than 10% to 15% are not associated with improved maternal or infant outcomes^1^. However, CS rates are increasing globally^2^. This rise has been particularly significant in Brazil, where the overall CS rate increased from 33% in 1991^3^ to 51.9% in 2012^4^. The high number of CS births in the privately funded healthcare sector makes a significant contribution to the high overall rates in Brazil, with CS rate as high as 80% in these settings^3^ ^,^ ^5^, independent of maternal or fetal complications during pregnancy or labour^5^.

The lack of routinely collected data in Brazil on CS clinical indications does not enable planned or emergency CS procedures to be considered separately. Maternal characteristics such as age, being overweight or obese at pregnancy commencement, and ethnicity^6^ ^,^ ^7^ do not explain variations in CS rates within or between countries. However, differences in criteria for fetal distress and dystocia^8^, organization of health care services^9^, and funding of maternity care^10^ have all been shown to influence CS rates. Therefore, an investigation of potential associations between maternal and obstetric variables and CS outcomes should also include how care was funded^10^. In Brazil, women access maternity care either through the unified national health system, the *Sistema Único de Saúde* (SUS), or through private healthcare providers. In 2012, nearly 25% of all births in Brazil were privately funded^5^. The payment to the clinician for assisting either a CS or a vaginal birth does not differ within the private and publicly funded systems, but a scheduled CS is time-saving and enables doctors to better organize their workload in private services^11^.

This study aimed to examine maternal and obstetric factors associated with CS births among women by primary source of health care funding (public or private).

## METHODS

A cross-sectional study was undertaken using data from the “Birth in Brazil” survey (*Nascer no Brasil*), conducted from February 2011 to July 2012 inclusively. This was the largest study to date to examine the impact of contemporary labor and birth management in Brazilian maternity settings on women’s health outcomes. Details on data collection and sampling methods have been described previously^12^ ^,^ ^13^.

Survey inclusion criteria included women with live infants (regardless of weight and gestational age) and women who had stillborn infants (weighing ≥ 500 g or gestational age ≥ 22 weeks) who gave birth in hospitals with 500 births or more in 2007. Women were excluded if they had a home birth, were unable to speak or read Portuguese, had a hearing impairment, severe mental illness, a termination of pregnancy, multiple pregnancies, or an instrumental birth (Figure).

**Figure:**
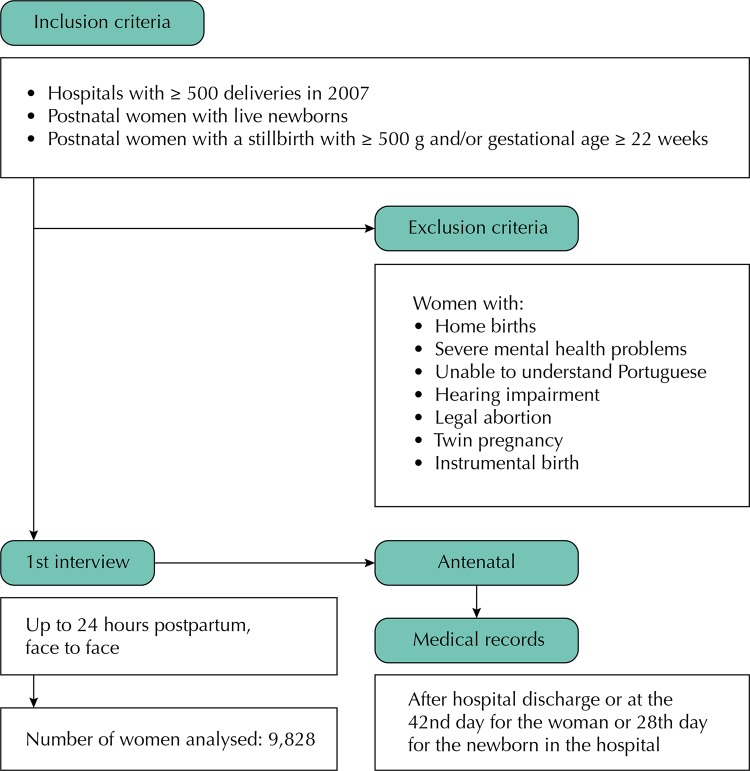
Data collection flowchart.

Six strata were created to calculate the sample size for each of the five macro-regions of the country: a capital or regional city hospital, and public, private or mixed funded hospitals (listed in the National Health Establishment Information System as private but with some beds contracted by SUS). In each stratum, random sampling was conducted in two stages: hospital and postnatal women. The sample size of each stratum was calculated based on CS prevalence in Brazil in 2007 (46.6%)^12^. A stratified random sample with proportional allocation took into account the numbers in each type of hospital selected. A 1.3 adjustment factor was used to calculate the size of cluster sampling, with each stratum estimated to provide between 444 to 450 women.

Ninety women were recruited from each hospital. In hospitals with fewer than 12 births a day, all women eligible were invited to participate. In hospitals with more than 12 births a day, women were selected using a sampling frame to ensure it included women who had given birth over the 24-hour period and on all days of the week.

Data collection included interviews with women prior to hospital postnatal discharge and routine data from obstetric and neonatal records. Information from women’s interviews was matched with their obstetric and neonatal records and antenatal charts (presented by 96.3% of women at the hospital admission)^14^ to supplement data that were frequently missing from these records. Women were followed up on day 28 (about their infant’s health) and day 42 (about their own health), including women transferred to other hospitals during labor or immediate postnatal period.

Only data from the Southeast region of Brazil are presented in this paper, which includes the states of São Paulo, Rio de Janeiro, Minas Gerais, and Espírito Santo and accounts for around 40% of the Brazilian population and the largest number of people with access to private healthcare. The region generates the largest gross income in Brazil. The main outcome variable in the study presented here was the mode of birth, classified as vaginal birth or CS. Analyses were conducted for these two groups, which were further classified into whether maternity care was privately or publicly funded.

We investigated maternal socioeconomic and demographic characteristics including age, skin colour (which is how ethnicity is recorded in Brazil), years in full-time education, marital status, employment status, economic status, location of the hospital, obstetric characteristics (previous CS and complications during index pregnancy or labour), and association with CS birth.

The rate of complications were estimated using composite variables based on maternal risk status and included one or more of the following, based on data from women’s interviews, antenatal charts, and maternal records: antenatal complications (including diabetes, chronic hypertension, cardiac disease, severe anaemia or other haemoglobinopathies, asthma, lupus or scleroderma, hyperthyroidism, chronic kidney disease, seizures or epilepsy, stroke, chronic liver disease, psychiatric disease, placenta praevia, abruption placenta, gestational hypertension, gestational diabetes, urinary infection, syphilis, HIV, and admission during pregnancy); and labor complications (including stroke, dystocia, dyskinesia, placenta praevia, meconium, fetal distress, preterm or post term gestation, restricted fetal growth, polyhydramnios or oligohydramnios, vaginal haemorrhage, and breech presentation).

Univariate and multivariate analysis were undertaken. Analyses were weighted to take account of the effect of the study design. For multivariate analysis, all variables were included in a binary logistic regression model using a stepwise forward process to enable comparisons between outcomes of interest and funding of care. In the final model, variables with a p < 0.05 were identified as independent predictors of CS. Planned or emergency CS procedures could not be differentiated, as these data were not routinely documented in either sector in women’s obstetric records. Because of this, the likelihood of a CS based on documented maternal or fetal complications was estimated using unadjusted and adjusted odds ratios (OR) with 95% confidence intervals (CI). Analyses were conducted using SPSS version 22 (IBM Corp., Amork, United States).

The “Birth in Brazil” survey was approved by the Research Ethics Committee of Escola Nacional de Saúde Pública, Fundação Oswaldo Cruz (Process 92/10) and by the Research Ethics Committee of Faculdade de Saúde Pública, Universidade de São Paulo (Process 717 944/14). All participants of the survey provided written informed consent.

## RESULTS

Mean maternal age was 26.3 years (SD = 6.4, ranged from 13 to 49 years). Women’s characteristics differed by how their maternity care was funded. Women whose care was publicly funded were more likely to be younger, of black or brown skin color, and from lower socioeconomic groups (C, D or E). They were more likely to have only attended elementary school, not to have a partner, and not to be in paid employment at pregnancy commencement. They were also more likely to have experienced pregnancy and labor complications in the index pregnancy and to not have had a previous CS (p < 0.001) (Table 1).

**Table 1:** Socioeconomic, demographic, clinical and obstetric characteristics of women who received intrapartum care funded by public or private health care. Southeastern Brazil, February 2011 to July 2012.

Characteristic	Public		Private		p^b^
	
	n	%	n	%	
Maternal age (years)					
12–19	1,487	19.7	128	5.7	< 0.001
20–34	5,396	71.3	1,716	76.0	
35 or more	683	9.0	415	18.3	
Total	7,566^a^	100	2,259	100	
Skin colour					
Indigenous	25	0.3	5	0.2	< 0.001
Black	835	11.0	95	4.2	
Brown	4,139	54.8	835	36.9	
Oriental	83	1,1	30	1,3	
White	2,482	32,8	1,294	57.3	
Total	7,564^a^	100	2,259	100	
Education					
Elementary school	4,172	55.3	330	14.7	< 0.001
High school	3,153	41.8	1,265	56.4	
College or more	220	2.9	649	28.9	
otal	7,545^a^	100	2,244^a^	100	
Marital status					
With partner	5,734	75.8	1,990	88.2	< 0.001
No partner	1,832	24.2	267	11.8	
Total	7,566^a^	100	2,257^a^	100	
Paid work					
No	4,790	63.3	674	29.8	< 0.001
Yes	2,777	36.7	1,585	70.2	
Total	7,567^a^	100	2,259	100	
Economic class					
A or B	1,376	18.3	1,416	63.5	< 0.001
C	4,780	63.5	770	34.5	
D or E	1,372	18.2	45	2.0	
Total	7,528^a^	100	2,231^a^	100	
Hospital location					
Capital	2,507	33.1	766	33.9	0.486
Regional	5,062	66.9	1,493	66.1	
Total	7,569	100	2,259	100	
Previous cesarian section					
No	2,603	34.4	350	15.5	< 0.001
Yes	1,608	21.2	653	28.9	
Primiparae	3,358	44.4	1,256	55.6	
Total	7,569	100	2,259	100	
Antenatal complications					
No	4,161	55.0	1,378	61.0	< 0.001
Yes	3,408	45.0	881	39.0	
Total	7,569	100	2,259	100	
Labour complications					
No	2,312	30.5	675	29.9	0.016
Yes	1,419	18.7	372	16.5	
No labour	3,838	50.8	1,212	53.6	
otal	7,569	100	2,259	100	

^a^ Missing < 10%.

^b^ Chi-squared test.

Birth data were available for 9,828 women, 77.0% of whom had maternity care provided in the public health system (n = 7,569) and 23.0% (n = 2,259) in the private sector. The overall CS rate was 53.0%, with a significantly higher proportion of CS among women who had private health care: 84.8% (1,916/2,259) *versus* 43.4% (3,288/7,569), p < 0.001 (Table 2). Indications for planned or unplanned CS were missing for 47.9% of women (4,863/10,156).

**Table 2:** Number and percentage of caesarean by characteristics of women and care funded from public or private health care. Southeastern Brazil, February 2011 to July 2012.

Funding source	Public				Private			
		
Variable	CS		Total	p*	CS		Total	p*
			
	n	%	n		n	%	n	
Total	3,288	43.4	7,569	-	1,916	84.8	2,259	< 0.001
Maternal age (years)								
12–19	499	33.5	1,488	< 0.001	80	62.5	128	< 0.001
20–34	2,431	45.1	5,396		1,465	85.4	1,716	
35 or more	358	52.4	683		371	89.4	415	
Skin colour								
Indigenous	8	32.0	25	< 0.001	4	66.7	6	< 0.001
Black	365	43.7	835		79	84.0	94	
Brown	1,686	40.7	4,139		663	79.5	834	
Oriental	34	41.0	83		24	82.8	29	
White	1,193	48.1	2,481		1,146	88.5	1,295	
Education								
Elementary school	1,647	39.5	4,172	< 0.001	236	71.5	330	< 0.001
High school	1,482	47.0	3,153		1,081	85.5	1,265	
College or more	149	67.7	220		588	90.6	649	
Marital status								
With partner	2,584	45.1	5,734	< 0.001	1,688	84.9	1,989	0,799
No partner	702	38.3	1,832		225	84.3	267	
Paid work								
No	1,943	40.6	4,791	< 0.001	494	73.3	674	< 0.001
Yes	1,344	48.4	2,777		1,422	89.7	1,585	
Economic class								
A and B	682	49.6	1.376	< 0.001	1,240	87.6	1,416	< 0.001
C	2,100	43.9	4,780		619	80.5	769	
D and E	489	35.7	1,371		32	71.1	45	
Hospital location								
Capital	2,423	47.9	5,061	< 0.001	1,311	87.8	1,493	< 0.001
Countryside	865	34.5	2,507		605	79.0	766	
Previous cesarian section								
No	450	17.3	2,603	< 0.001	168	48.0	350	< 0.001
Yes	1,311	81.5	1,608		641	98.2	653	
Primiparae	1,526	45.5	3,357		1,107	88.1	1,256	
Antenatal complications								
No	1,548	37.2	4,161	< 0.001	1,143	82.9	1,378	0.002
Yes	1,740	51.1	3,408		773	87.7	881	
Labour complications								
No	747	32.3	2,312	< 0.001	561	83.2	674	< 0.001
Yes	846	59.6	1,419		346	93.0	372	
No labour	1,695	44.2	3,838		1,009	83.2	1,213	

* Chi-squared test.

In contrast, women whose care was funded privately were more likely to be white and over 35 years of age, from higher socioeconomic groups (A or B) and to be primiparous. They were more likely to have attended college or have more education, to have a partner, to be in paid employment, and to have had a previous CS (p < 0.001) (Table 1). The only variable that was not associated with CS in the private sector was marital status (Table 2).

In the adjusted multivariate analysis (Table 3), reduced likelihood of a CS birth in either sector was associated with younger maternal age and giving birth in a hospital located in a state capital city. Not having a partner was also associated with a lower likelihood of CS, but only in the public system. In contrast, having a CS in the publicly funded system was associated with older maternal age, more years in full-time education, being in paid employment, higher socioeconomic status, a previous CS, primiparity, and complications in the index pregnancy or labor. In the private sector, paid employment, a previous CS, primiparity, and complications in the index pregnancy or labor were associated with a CS.

**Table 3:** Crude and adjusted odds ratio, 95% confidence intervals (95%CI) for variables associated with caesarean funded by public or private health care. Southeastern Brazil, February 2011 to July 2012.

Funding source	Public				Private			
		
Variable	OR crude	95%CI	OR adjusted*	95%CI	OR crude	95%CI	OR adjusted*	95%CI
Maternal age (years)								
12–19	0.61	0.55–0.69	**0.63**	**0.54–0.73**	0.28	0.19–0.42	**0.39**	**0.23–0.66**
20–34	1.00	-	1.00	-	1.00	-	Ref.	-
35 or more	1.34	1.14–1.57	**1.44**	**1.16–1.77**	1.45	1.03–2.04	1.54	1.00–2.37
Skin colour								
Indigenous	1.00	-	1.00	-	1.00	-	1.00	-
Black	1.70	0.73–3.97	2.53	0.94–6.80	2.70	0.42–17.27	4.34	0.52–34.84
Brown	1.50	0.65–3.48	1.84	0.69–4.90	2.03	0.34–12.03	3.56	0.47–26.81
Oriental	1.51	0.59–3.89	1.63	0.54–4.93	2.38	0.32–17.66	2.46	0.26–23.43
White	2.20	0.87–4.69	2.37	0.89–6.31	4.03	0.68–23.90	4.76	0.63–35.71
Education								
Elementary school	1.00	-	1.00	-	1.00	-	1.00	-
High school	1.36	1.24–1.49	0.96	0.849–1.092	2.33	1.75–3.11	0.94	0.63–1.40
College or more	3.23	2.41–4.31	**1.63**	**1.15–2.31**	3.82	2.68–5.46	0.90	0.53–1.52
Marital status								
With partner	1.00	-	1.00	-	1.00	-	1.00	-
No partner	0.76	0.68–0.84	**0.81**	**0.71–0.92**	0.92	0.68–1.37	0.98	0.64–1.50
Paid work								
No	1.00	-	1.00	-	1.00	-	1.00	-
Yes	1.37	1.25–1.51	**1.32**	**1.17–1.49**	3.18	2.51–4.03	**2.70**	**1.99–3.66**
Economic class								
A or B	1.78	1.52–2.06	**1.52**	**1.25–1.85**	2.80	1.44–5.45	2.11	0.94–4.75
C	1.41	1.25–1.60	**1.36**	**1.17–1.58**	1.63	0.84–3.19	2.12	0.94–4.75
D or E	1.00	-	1.00	-	1.00	-	1.00	-
Hospital location								
Capital	0.57	0.52–0.63	**0.49**	**0.44–0.56**	0.52	0.41–0.65	**0.49**	**0.37–0.65**
Regional	1.00	-	1.00	-	1.00	-	1.00	-
Previous cesarian section								
No	1.00	-	1.00	-	1.00	-	1.00.	-
Yes	21.13	17.97–24.85	**22.46**	**18.94–26.64**	59.78	32.28–110.71	**58.22**	**30.78–110.13**
Primiparae	3.98	3.53–4.50	**4.74**	**4.11–5.47**	8.07	6.16–10.57	**8.64**	**6.20–12.02**
Antenatal complications								
No	1.00	-	1.00	-	1.00	-	1.00	-
Yes	1.76	1.61–1.93	**1.60**	**1.43–1.78**	1.45	1.15–1.87	**1.39**	**1.03–1.87**
Labour complications								
No	0.60	0.54–0.67	0.58	0.51–0.66	1.00	0.77–1.28	0.88	0.65–1.20
Yes	1.86	1.65–2.11	**1.99**	**1.72–2.30**	2.69	1.76–4.13	**3.56**	**2.16–5.93**
No labour	1.00	-	1.00	-	1.00	-	1.00	-

Values with statistical significance are shown in bold.

* Models adjusted for all variables presented in this table.

Although some of the factors associated with a CS were common regardless of the source of health care funding, the magnitude of the effect differed. For example, women who had a previous CS were almost three times more likely to have another CS in the index pregnancy if their care was provided in the private sector (Table 3).

## DISCUSSION

This is the first study to examine associations between maternal and obstetric characteristics, maternity funding, and impact on caesarean births in Southeast Brazil. There was an association between higher socioeconomic status, obstetric factors and CS births among women whose maternity care was publicly funded. Conversely, most maternal socioeconomic factors explored were not associated with a CS in the private sector, as most women in this sector were of higher socioeconomic status and had undergone extremely high rates of surgery.

Limitations include that indications associated with the medical need for CS birth could not be discriminated in the analyses, as these data were not routinely recorded. However, the high CS rate in our study, which was powered to represent the population of women giving birth in Southeastern Brazil, suggests that a significant number of operations was performed without medical indication in both sectors^15^. Study strengths include that variables incorporated information from maternal and neonatal records and postnatal interviews with women to verify and correct inconsistencies in routinely recorded data.

In the private health sector, few women may be offered the option of achieving a vaginal birth. This particular aspect of Brazilian maternity culture impairs examination of factors, such as failure to progress, fetal compromise or fetal malpresentation commonly associated with a higher CS rate^11^ ^,^ ^16^. More than half of all women in both sectors had a pre-labor CS and 92% of these women had a late preterm baby^4^, which is likely to be detrimental to maternal and infant health and reflects a system of care that does not promote maternal choice with respect to normal birth.

The association between CS rate and private health care reflects findings from previous studies in Brazil^17^, United States^18^, and China^19^. A study that investigated CS rates in Peru showed an increase from 1991 to 1999 and from 1999 to 2008 of 24.5% in the privately funded sector and under 8% in the public sector^20^.

Factors including wide spread coverage of private healthcare, pregnancy, and birth care provided almost entirely by obstetricians (with labor and birth care provided by midwives or nurse-midwives ranging from 3% to 20%^11^) and convenience of scheduling surgery^21^ ^,^ ^22^ are possible explanations. Although payment for CS and vaginal births does not differ between private and public sectors, women who have spontaneous labor are likely to require clinical care over an undefined number of hours (unlike a scheduled CS). This condition has a strong influence on CS rates in Brazil^11^.

Higher socioeconomic status and university attendance did not influence CS rates in the private sector, as most women belonged to these groups. Despite the strong influence of these variables on CS section rates, the funding source was a decisive influence on the mode of birth among these women, independently of risk, a finding also reported in previous studies from Brazil^10^ ^,^ ^11^. Younger women were less likely to have a CS in the current study particularly in the public health system, presumably as they were less likely to have private healthcare and possibly more likely to have the support of a birth companion than older women in the study, with robust evidence that a birth companion reduces labor interventions^9^. As a first CS birth can negatively affect future maternity outcomes^16^, maternal age could have influenced decisions to progress to a CS.

Findings support previous studies that CS rates increase with maternal age^23^. Factors commonly reported among older women (such as a previous CS) and increased risk of medical complications (such as hypertension or diabetes) offer potential justification for opting for a CS^5^. This is of concern for women, maternity care providers, and funders in many countries, with the current trend for women to delay their first pregnancy^23^.

Although years in full-time education and higher socioeconomic status were associated with increased likelihood of CS in the public sector, women’s views suggest that a CS would not be a better first choice^25^. Women who did not have a partner were less likely to have a CS, but only if care was publicly funded, in which younger maternal age, being in a lower socioeconomic group, and having had a previous normal vaginal birth also influenced outcomes. Because these women were also unlikely to have paid for private maternity care, as described earlier, their chance of CS was reduced still further^10^.

The association with decreased CS in state capitals of the Southeastern Brazil regardless of funding source may reflect the ability of maternity care providers in these settings to implement evidence-based guidance to support normal birth^26^, although the proportion of CS births was smaller following bivariate analysis. Reasons are likely to be complex, but some factors may have inverted the trend of the likelihood for a CS birth, such as women being more likely to have information to inform decision making, and evidence that regional hospitals are facing cuts to resources including qualified clinicians and are less likely to promote the implementation of evidence-based practice^27^.

Nevertheless, despite evidence that vaginal birth after a caesarean section is a safe alternative for women who had a previous CS^28^, it did not appear to have been routinely offered to women in either sector. Although primiparous women had an increased chance of a CS regardless of maternity care funding, it was more likely to be offered to women who had private health care, despite recommendations to avoid a first CS^16^. Primiparous women have longer labors and robust data has shown^29^ that progress of labor is slower than originally proposed by Friedman, although the latter’s work is still likely to influence decisions about progression to a CS.

Complications during pregnancy also increased the chance of a CS regardless of how care was funded. Women whose care was provided by the public system may have had more serious problems requiring intervention^30^. Even after adjusting for other potential confounders, there was no association with pregnancy complications in women who did not go into labor in the private care sector, possibly as data on too few women were available^31^.

Rates of CS were high in the Southeast region of Brazil, especially among women who had private health care, with socioeconomic status being an important predictor of CS. The regulation of obstetric practice in privately funded care is urgently needed, given that this is a sector that provides maternity care for a large proportion of women in this region. Qualitative studies are also needed to better understand reasons for CS among women of higher socioeconomic status who receive publicly funded care. Measures to support normal birth and better information on birth choices for women and their families are essential to encourage informed choice and decision making on benefits and consequences of the mode of birth for all women giving birth in Brazil.
